# Coordination-Driven Nanoarchitectures for Smart Packaging Strategies in Poultry Preservation

**DOI:** 10.34133/research.0981

**Published:** 2026-01-15

**Authors:** Tianyi Ma, Jianing Yang, Yunbo Luo, Xin Zhou, Jinxuan Cao, Hao Zhang, Ying Wang, Nan Cheng

**Affiliations:** ^1^ Beijing Laboratory for Food Quality and Safety, College of Food Science and Nutritional Engineering, China Agricultural University, Beijing 100083, P. R. China.; ^2^ Key Laboratory of Geriatric Nutrition and Health (Beijing Technology and Business University), Ministry of Education, Beijing 100048, P. R. China.; ^3^ Beijing Engineering and Technology Research Center of Food Additives, School of Food and Health, Beijing Technology and Business University, Beijing 100048, P. R. China.; ^4^ College of Chemistry and Chemical Engineering, Shandong Sino-Japanese Center for Collaborative Research of Carbon Nanomaterials, Laboratory of Fiber Materials and Modern Textile, The Growing Base for State Key Laboratory, Qingdao University, Shandong 266071, P. R. China.; ^5^ Key Laboratory of Safety Assessment of Genetically Modified Organism (Food Safety), Ministry of Agriculture, Beijing 100083, P. R. China.

## Abstract

The poultry industry faces major challenges in preserving meat freshness and safety due to high water activity, rapid microbial growth, and oxidative spoilage. Traditional methods such as vacuum sealing and antioxidants are insufficient, as they cannot effectively suppress anaerobic pathogens and lack real-time freshness assessment. This review introduces a transformative strategy that applies coordination chemistry to design multifunctional nanomaterials for poultry preservation. Dynamic metal–ligand interactions—including redox-active centers, stimuli-responsive bonds, and host–guest adsorption—allow precise antibacterial control through 4 mechanisms: ligand-regulated ion release, reactive oxygen species (ROS) generation, coordination-triggered antimicrobial delivery, and electrostatic membrane disruption. In addition, freshness can be monitored by biomarker-specific coordination responses, such as nanoparticle aggregation for optical signals or MOF (metal–organic framework)-based volatile amine capture for colorimetric and electrochemical detection. Integration with oxygen scavengers, humidity regulators, and pH-responsive systems optimizes the packaging environment. Coupling with digital technologies further enables intelligent platforms for autonomous quality validation and supply chain transparency. This approach connects molecular-scale coordination principles with engineering practice while addressing biodegradability, environmental resilience, and scalability to reduce waste and achieve sustainable poultry preservation.

## Introduction

The global poultry industry, an essential pillar of food security, faces increasing challenges in maintaining the freshness and safety of its products due to the unique biochemical properties of poultry meat. For example, in WHO (World Health Organization)/FAO (Food and Agriculture Organization) burden-of-disease estimates, *Campylobacter* spp. and nontyphoidal *Salmonella* combined cause over 95 million illnesses annually, and commercial poultry meat is identified as a major vehicle of transmission [1]. In retail surveys, contamination with Campylobacter in poultry has been detected at rates up to ~45.8% in Japan and ~63% in Iran [2,3]. Poultry, characterized by high water activity, unstable myoglobin REDOX (reduction–oxidation) states, and a high abundance of polyunsaturated fatty acids, is more susceptible to accelerated microbial growth and oxidative spoilage compared to red meats like beef and pork [4]. Traditional packaging methods for fresh poultry, such as antioxidant application and vacuum sealing, are limited in preventing anaerobic bacterial growth and rely heavily on cold storage. Additionally, the lack of real-time visual indicators for freshness contributes to food safety risks and avoidable waste. In the meat sector, it is estimated that ~23% of production is lost or wasted over the supply chain—from farm to consumer—with an important fraction attributable to spoilage and quality degradation [5]. Such spoilage-driven loss not only imposes economic burdens but also undermines sustainability and trust in the poultry industry [6].

In this context, coordination chemistry is now a transformative approach for the rational design of nanomaterials with engineered functions [7,8]. By harnessing metal–ligand interactions—including redox-active metal centers and dynamic coordination networks—researchers can construct multifunctional systems capable of delivering antimicrobial effects and enabling molecular-level condition monitoring for poultry spoilage [9]. These new methods for preserving fresh poultry meat redefine preservation strategies through 3 interconnected aspects: (a) inhibition of bacterial growth on the surface of fresh poultry meat using metal ion release, photocatalytic reactive oxygen species (ROS) production, or the physical bactericidal action of nanomaterials; (b) monitoring freshness by analyzing nanomaterial–ligand interactions and biomarker-specific coordination complexes; (c) regulation of the packaging microenvironment, including oxygen scavenging, humidity balance, and pH stabilization, to slow biochemical spoilage processes [9].

Although some studies have explored the application of nanomaterials guided by coordination chemistry principles for food preservation, a comprehensive packaging strategy for fresh poultry remains absent. Such solutions should seamlessly integrate antimicrobial functionality, freshness preservation, and real-time monitoring. Current research predominantly focuses on individual material properties, often overlooking the synergistic integration of preservation, sensing, and packaging engineering. Furthermore, the poultry-specific adaptation of nanomaterials—considering its high-humidity microenvironment, pH-dependent myoglobin denaturation, and lipid composition—warrants more systematic investigation.

Unlike previous general reviews on food nanotechnology that mainly provide broad overviews of material classes and mechanisms, this review is uniquely positioned by focusing on the poultry matrix and by integrating antibacterial mechanisms, freshness monitoring, and packaging design within a coordination chemistry framework [10–12]. This review addresses these issues by combining them to build next-generation smart packaging based on nano-coordination chemistry. We first systematically discussed the biochemical and microbial specificity of poultry spoilage and compared it with the degradation pathway of red meat. Next, we systematically divide the functions of nanomaterials into 3 modules consistent with the following sections: antibacterial mechanisms via coordination chemistry, freshness monitoring via coordination-based sensing, and microenvironment regulation strategies. The antimicrobial mechanisms are classified into coordination-controlled antimicrobial release, band-gap-engineered photocatalysis, and ligand-directed nanostructures based on the coordination of chemically driven processes. At the same time, freshness monitoring strategies were analyzed through nanomaterial-based ligand analysis identification to highlight the biomarker-specific coordination response.

Crucially, we explore emerging nanomaterials that integrate active preservation methods such as oxygen removal and humidity control through a dynamic coordinated network, addressing poultry’s high-water activity and oxidation vulnerability. In addition to material innovation, we look forward to coordinating the application of engineered film with RFID (radio frequency identification devices) and QR code (quick response code) technology in fresh poultry packaging for real-time traceability and blockchain-compatible supply chain management.

**Fig. 1.**
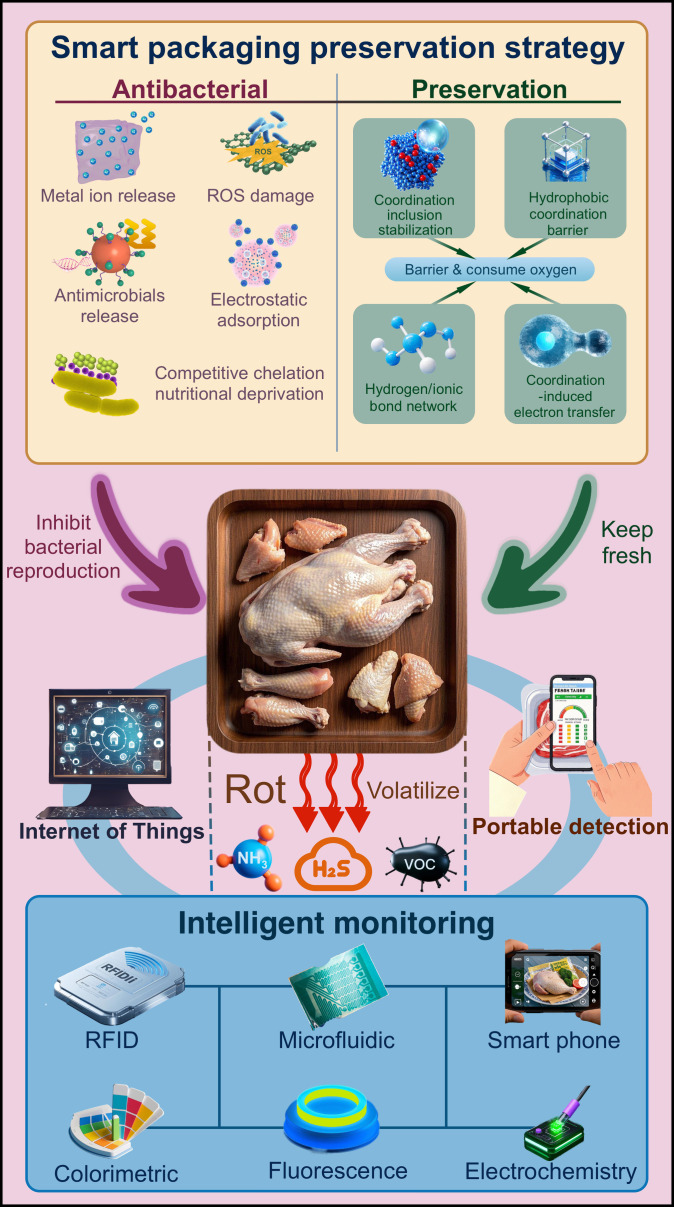
An overview of the coordination chemical mechanisms of nanomaterials in fresh poultry meat preservation, antibacterial, and monitoring.

This review offers a transformative perspective on poultry preservation by connecting molecular-scale coordination principles with macroscale food engineering. It synthesizes recent advances and highlights underexplored intersections among coordination chemistry, nanotechnology, and digital food systems, thereby laying the groundwork for intelligent, self-validating preservation platforms (Fig. 1).

## Specific Spoilage Pathways of Poultry Meat

Poultry spoilage constitutes a complex biochemical network influenced by multiple interrelated factors, including microbial metabolism, endogenous enzymatic activity, and oxidative reactions. The spoilage pathways are species-specific and predominantly involve the preferential degradation of phospholipids, accompanied by the generation of characteristic odors resulting from these processes.

In the process of poultry spoilage, *Pseudomonas*, *Shewanella*, and *Enterobacteriaceae* are generally regarded as the dominant bacterial groups [13]. Their metabolic activities generate key spoilage-associated metabolites that directly affect both sensory quality and safety. For example, *Pseudomonas* spp. degrades amino acids and lipids to produce volatile sulfur compounds and ketones, which cause strong off-odors and surface slime formation [14]. *Shewanella* spp. contribute to trimethylamine and putrescine accumulation, leading to fishy or ammonia-like odors and potential histamine hazards [15]. Members of the Enterobacteriaceae family produce biogenic amines, lactic acids, and acetic acids, which not only alter flavor but also may pose food safety concerns at elevated concentrations [16].

In addition to spoilage caused by microorganisms, the natural enzymatic activity in poultry meat plays a key role. After slaughter, an increase in calcium ion (Ca^2+^) concentration activates the enzyme μ-calpain [17]. This enzyme specifically cleaves proteins such as desman and titin, which leads to the breakdown of the muscle structure [18,19]. As a result, sarcoplasmic proteins like myoglobin and creatine kinase are released into the extracellular matrix. This release provides a source of nitrogen for microorganisms, creating a positive feedback loop that promotes their further growth and reproduction [20].

In addition, the muscle fiber diameter of poultry meat is usually 30 to 50 μm, which is smaller than the 50 to 100 μm of beef, and the relative surface area is more extensive, further accelerating the invasion of microorganisms [21]. In addition, the connective tissue content of poultry meat is low, and its collagen only accounts for 2% of its total protein, which limits the spatial colonization resistance of spoilage bacteria [22]. Moreover, the glutathione peroxidase (GPx) activity in poultry has been reported to be approximately 40% lower than that in red meat, weakening its ROS clearance capacity [23]. This biochemical deficiency accelerates lipid oxidation, as reflected by markedly higher TBARS (thiobarbituric acid reactive substances) values in poultry meat (e.g., 1.8 to 2.3 mg MDA (malondialdehyde)/kg after 5 d at 4 °C) compared to beef (0.9 to 1.2 mg MDA/kg) [16,24,25]. In parallel, poultry meat accumulates higher levels of volatile aldehydes such as hexanal and nonanal, which are key contributors to rancid off-flavors [26]. Spoilage progression is also faster in poultry than in red meat, as demonstrated by microbiological and chemical indices. Comparative studies show that poultry meat typically reaches the TVB-N (total volatile basic nitrogen) spoilage threshold of 25 to 30 mg/100 g within 5 to 6 d at refrigeration, whereas beef requires 9 to 12 d [27]. Similarly, total bacterial counts (TBCs) in poultry exceed 10^7^ colony-forming units (CFU)/g in less than a week under chilled storage, about 1.5 to 2 times faster than red meat [24]. These data collectively substantiate the conclusion that poultry meat is more prone to rapid deterioration.

**Fig. 2.**
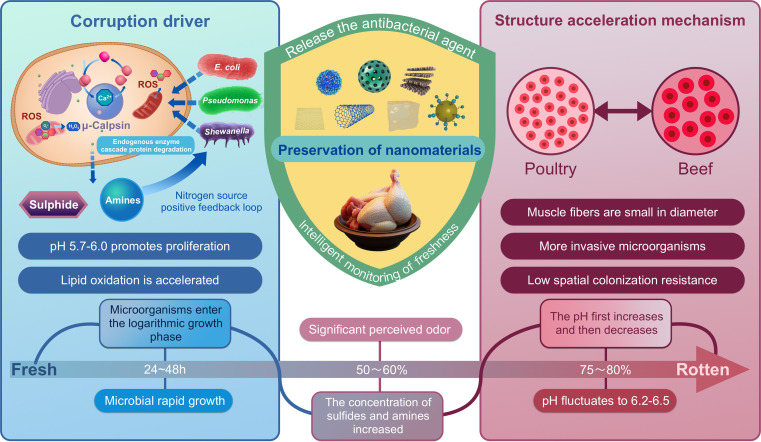
Specific spoilage pathways of poultry meat.

For effective and efficient preservation of fresh poultry meat, nanomaterials based on coordination chemistry are expected to achieve multi-dimensional innovation in the packaging of fresh poultry meat. In terms of microbial regulation, functionalized nanoparticles were targeted to inhibit the quorum sensing system of spoilage bacteria and the activity of key metabolic enzymes, while nano-capsules were used to slow-release natural antimicrobials to block biofilm formation [28]. Targeting the endogenous enzyme-mediated release of nitrogen sources, a nano-system with localized activity and ion-chelating capability, has been developed to precisely regulate calcium homeostasis and retard the muscle autolysis process. In constructing an antioxidant barrier, biomimetic nanozymes scavenge free radicals by mimicking endogenous antioxidant mechanisms, whereas nanocomposite barrier materials impede oxygen penetration through multi-scale structural design. Current research primarily addresses the migration safety of nanomaterials, elucidates multi-target synergistic mechanisms, and explores the integration of such systems with other preservation technologies. The future development direction will focus on the bionic design of degradable nano-capsules, the development of dynamic, responsive, intelligent monitoring systems, and the construction of the integrated technology system of “activity regulation and real-time warning” based on the spoilage molecule network [29]. It provides theoretical support and a technical paradigm for realizing loss reduction in the poultry supply chain (Fig. 2).

The coordination of chemistry-based nanomaterials is expected to achieve multidimensional innovations in fresh poultry meat packaging for effective and efficient preservation. In microbial regulation, functionalized nanoparticles demonstrate targeted inhibition of quorum sensing systems and key metabolic enzyme activities in spoilage bacteria, while nano-carriers enable sustained release of natural antimicrobial agents to disrupt biofilm formation. Current research priorities include the safety assessment of nanomaterial migration, the elucidation of multi-target synergistic mechanisms, and the systematic integration with complementary preservation technologies. Future directions are expected to focus on the biomimetic design of degradable nanocarriers, the development of stimuli-responsive intelligent monitoring systems, and the establishment of an integrated technological framework that couples “activity regulation” with real-time warning based on spoilage-related molecular networks. These advancements provide the theoretical foundations and technological paradigms for reducing losses in poultry supply chains.

## Antibacterial Mechanisms via Coordination Chemistry

Antibacterial mechanisms based on coordination chemistry have become a cornerstone in nanomaterial-enabled poultry preservation. These strategies exploit the interplay between metal ions, ligands, and bacterial biomolecules, enabling precise disruption of microbial structures and functions. Compared with conventional preservatives, coordination-driven systems offer sustained efficacy and environmental responsiveness.

**Fig. 3.**
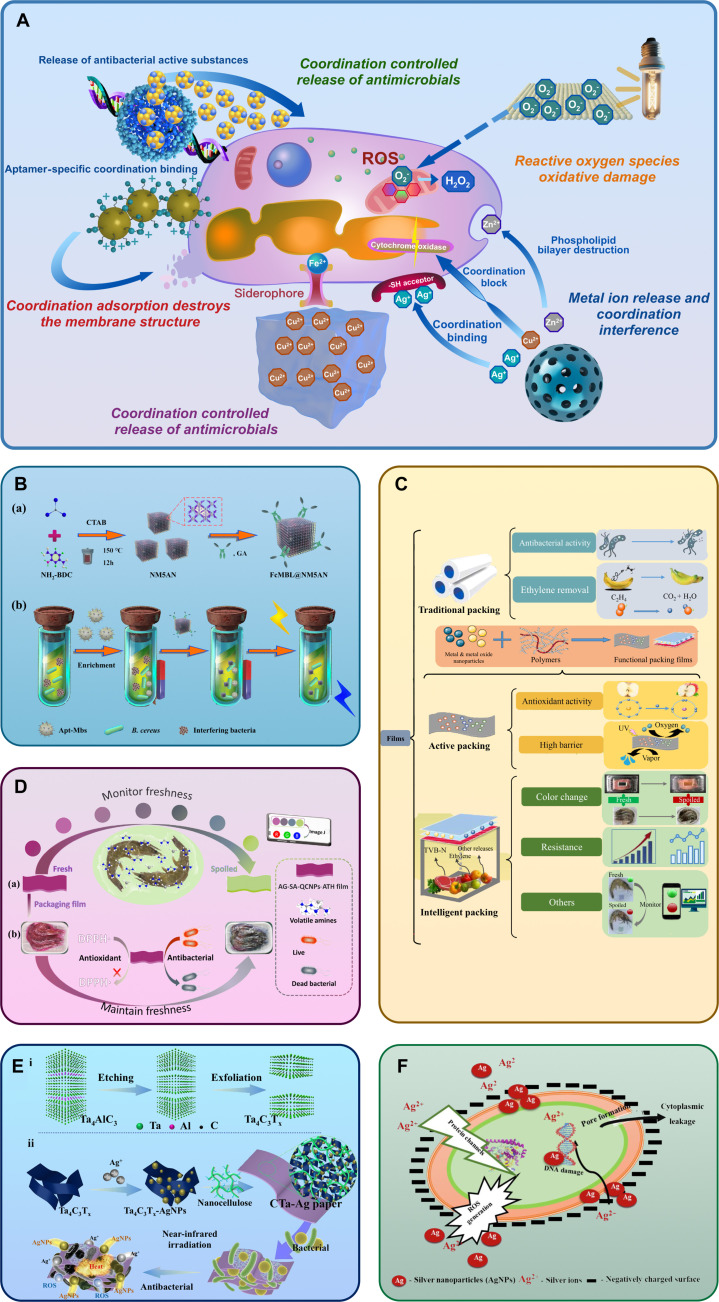
Antibacterial mechanisms via coordination chemistry. (A) Antibacterial mechanism mediated by coordination nanomaterials. (B) Preparation of FcMBL@NM5AN and determination of *Bacillus cereus* in mixtures using the dual recognition strategy [93]. (C) Principles and applications of traditional packaging, active packaging, and intelligent packaging [94]. (D) Schematic diagram of colorimetric monitoring of shrimp freshness based on multifunctional film combined with smartphone (a) and application of multifunctional film in shrimp preservation (b) [40]. (E) Schematic illustration for the fabrication of CTa-Ag packaging [95]. (F) Mechanism involved in the bactericidal activity of AgNPs due to its nano and ionic attributes. DNA, deoxyribonucleic acid; ROS, reactive oxygen species [96].

Although many antimicrobial mechanisms have been validated in beef, pork, or dairy matrices, several studies have also directly examined poultry systems. For instance, ZnO–chitosan nanocomposite coatings applied to chicken breast fillets effectively reduced *Escherichia coli* and *Staphylococcus aureus* populations by over 2 log CFU/g during 7 d of refrigeration while extending sensory acceptability by 3 to 4 d (Fig. 3 and Table 1).

**Table 1.** Antibacterial mechanisms via coordination chemistry

Nanomaterial	Mechanism	Coordination	Detection target	Bacteria	Antibacterial effect	Carrier	Reference
AgTi NPs	Metal ion release and coordination interference​	-SH coordination	Water	*E. coli*	Almost 100% (3 h contact with *E. coli*)	Polyvinylidene fluoride (PVDF) membrane	[97]
Fe-doped titanite	Reactive oxygen species oxidative damage	Fe^2+^ and Fe^3+^ coordination	Medical implant	*S. aureus* and *E. coli*	Sterilization rate: 90% (*S. aureus*), 91% (*E. coli*), 93% (MRSA)	Fe-doped titanite	[98]
CTS-TiO_2_-Ag	Metal ion release and coordination interference	C=O coordination	Potato and strawberry	*E. coli*, *S. aureus*, *Bacillus subtilis*	Antibacterial zone diameter: 22.00 mm (*E. coli*); antibacterial zone diameter: 20.33 mm (*S. aureus*)	Chitosan	[99]
V-doped TiO_2_	Reactive oxygen species oxidative damage	ROS	Fresh raw milk	Mesophilic aerobic and facultative anaerobic microorganisms (MAnFAM)	The antibacterial rate of 3, 5, and 9 d was 60%, 75%, and 80%, respectively (MAnFAM).	Polypropylene (PP)	[100]
Nano-ZnO	Reactive oxygen species oxidative damage	Coordination releases metal ions	LLDPE and PC	*E. coli* and *S. aureus*	Greater than 99.9% (*E. coli, S. aureus*)	LLDPE and PC	[101]
Ch-CNE and Ch-RNE	Coordination adsorption destroys the membrane structure	Coordination chelation	Ground meat	*Listeria monocytogenes* and *Salmonella enterica serovar* Typhimurium	The quantity decreased by 1 log_10_ (CFU/g) (*L. monocytogenes*, Ch-RNE, 7 d) and the quantity decreased by 3 log_10_ (CFU/g) (*S. enterica serovar* Typhimurium, Ch-RNE, 12 d).	Chitosan	[102]
Ag@SiO_2_	Coordination controlled release of antimicrobials	-SH coordination	Beef patties	*E. coli* and *S. aureus*	Almost 100% (*E. coli*, 0.24% nanoparticle content, 6 h)	SiO_2_	[31]
ZnO-Nps	Coordination adsorption destroys the membrane structure	ROS	Carboxymethyl cellulose/gelatin hydrogel films	*S. aureus, B. subtilis, L. monocytogenes, Enterobacter aerogenase, E. coli, Bordetella bronchiseptica*	Antibacterial zone diameter: 30 ± 1.03 mm (*S. aureus*); inhibition zone diameter: 31 ± 1.25 mm (*E. coli*)	Carboxymethyl cellulose/gelatin hydrogel films	[33]
Nano-ZnO	Coordination adsorption destroys the membrane structure	Zn^2+^ coordination	Pork	*E. coli* and *S. aureus*	Antibacterial rate: 64.58% (*E. coli*); antibacterial rate: 85.92% (*S. aureus*)	Yam starch/microcrystalline cellulose (SC)	[103]
Metal-phenolic networks (MPN)	Coordination adsorption destroys the membrane structure	Fe^3+^ coordination	Pork	*E. coli* and *S. aureus*	Antibacterial rate: 99% (*E. coli*); antibacterial rate: 98% (*S. aureus*)	Sodium alginate (SA)	[104]
ZC and ZCT	Reactive oxygen species oxidative damage	Imine bond coordination	Pork	*E. coli* and *S. aureus*	Antibacterial zone diameter: 18.39 ± 1.22 mm (*S. aureus*, CA); antibacterial zone diameter: 15.70 ± 1.75 mm (*E. coli*, CA)	Pickering emulsions	[105]
Nano-ZnO	Coordination adsorption destroys the membrane structure	-	Fresh meat	*S. aureus* and *E. coli*	Reach 100% (*E. coli*, *S. aureus*, 2 h)	PET/SiO*_x_*	[106]
Chitosan nanoparticles (CNPs)	Coordination adsorption destroys the membrane structure	-NH_2_ coordination	Ground meat and Kashar cheese	*S. aureus* and *E. coli*	Sterilization rate was about 95% (*E. coli*, 25 °C, 6 d), sterilization rate was about 99% (*S. aureus*, 25 °C, 6 d).	Whey protein concentrate and hydroxypropyl methylcellulose	[107]
Quercetin-loaded chitosan nanoparticles	Coordination adsorption destroys the membrane structure	-NH_2_ and -OH coordination	Shrimp	*S. aureus* and *E. coli*	Bactericidal rate increased by 84.16% (*S. aureus*) and 38.39% (*E. coli*)	Chitosan	[40]
ZnO NPs	Coordination adsorption destroys the membrane structure	-SH and -COOH coordination	Food packaging	*S. aureus*, *E. coli*, *B. subtilis*	>95% (*S. aureus* and *B. subtilis*)	Thermoplastic chitosan	[38]
Silver nano-inks	Metal ion release and coordination interference	-SH, -COOH and -NH_2_ coordination	Food packaging	*S. aureus* and *E. coli*	The size of the inhibition zone is 6.45 mm (*E. coli*)	Cyclo-olefin polymer	[96]
Self-assembled carboxymethyl chitosan/zinc alginate composite film	Metal ion release and coordination interference	-COOH coordination	Chilled pork	*S. aureus* and *E. coli*	Refrigerated shelf life of 6 d	CMCS and SA composite membrane	[108]

### Metal ion release and coordination interference

Controlled release of Ag^+^, Zn^2+^, and Cu^2+^ ions from coordination nanomaterials provides broad-spectrum antibacterial activity [30]. These ions coordinate with key bacterial biomolecules such as enzymes, DNA, and membrane proteins, disrupting their function.

For instance, Ag@SiO₂ nanoparticles regulate Ag^+^ release through the silica shell, ensuring long-term activity while avoiding sudden toxicity peaks [31]. The released Ag^+^ binds to sulfhydryl groups in bacterial membrane proteins, impairing respiratory enzyme activity, while Cu^2+^ can coordinate with histidine residues in cytochrome c oxidase, blocking electron transfer [32]. Synergistic bimetallic systems further enhance efficiency by targeting multiple pathways simultaneously [33].

These systems are effective against both Gram-positive and Gram-negative bacteria in meat matrices and can be tailored by mesoporous carriers or MOFs to match ion release rates with poultry spoilage kinetics. However, excessive ion release risks interacting with meat proteins such as myoglobin, potentially causing discoloration, underscoring the need for controlled kinetics.

### ROS oxidative damage

ROS generation is another key antibacterial pathway. Nanomaterials with tailored coordination environments enhance the production of ·OH and ·O₂^−^ radicals, which oxidize lipids, proteins, and DNA in bacterial cells [34,35].

A notable example is the CuBSA coordination nanozyme, which utilizes the Cu^2+^/Cu^+^ redox cycle to generate hydroxyl radicals, achieving >95% bactericidal activity against *E. coli* and *S. aureus*. Packaging films embedding photocatalytic nanomaterials such as PVA/g-C₃N₄ further demonstrate >99% inhibition of bacteria while simultaneously reducing spoilage markers such as TVB-N in meat [36].

The ROS pathway offers broad-spectrum antibacterial properties but requires careful evaluation of nanomaterial migration and potential long-term toxicity. Future directions include developing light-independent or self-powered ROS systems to enhance applicability in poultry cold chain environments.

### Coordination controlled release of antimicrobials

By harnessing metal–ligand bonds, nanomaterials can achieve stimuli-responsive release of antimicrobials. This mechanism reduces nonspecific toxicity and enables targeted action in response to environmental changes such as pH or bacterial metabolites [37].

In 2022, Zhang et al. [38] constructed a chitosan/nano-zone (CZ) composite that is capable of constructing a stable network through the amino-Zn^2+^ coordination. This coordination structure not only improves thermal stability but also achieves the slow release of Zn^2+^. In this system, chitosan adsorbs the bacterial membrane by positive charge, and Zn^2+^ destroys the membrane integrity. The experiment on chicken preservation showed that CZ5 film containing 5% ZnO reduced the number of pseudomonas by 2.3 log CFU/g, lipid oxidation value by 42%, and shelf life by 5 d. Similarly, DNA–AgNC aptamer systems use aptamer conformational changes to regulate Ag^+^ release, achieving a 95% inhibition rate against *S. aureus* [39], providing direct evidence of the effectiveness of coordination-driven ZnO composites in poultry packaging.

Such systems exemplify how coordination chemistry can link molecular recognition with coordination-controlled antimicrobial release, paving the way for “intelligent” antimicrobial packaging.

### Coordination adsorption destroys the membrane structure

Electrostatic attraction between positively charged coordination nanomaterials and negatively charged bacterial membranes results in physical damage [37].

Quercetin-loaded chitosan nanoparticles (QCNPs), for instance, adhere to bacterial membranes via protonated amino groups, disrupting lipid bilayers. The encapsulated quercetin further intercalates into fatty acid chains, destabilizing membrane integrity. In shrimp preservation, this system effectively reduced bacterial counts and lipid oxidation, demonstrating dual antibacterial and antioxidant activity that could be adapted to poultry packaging [40].

Silver nanoparticles synthesized in situ using carboxymethyl cellulose also show dual pathways: Ag^+^ coordination with sulfhydryl groups and direct electrostatic disruption. This strategy reduces total viable counts in beef by >2 log CFU/g, illustrating broad applicability across meat systems [41].

### Nutrient deprivation through competitive chelation

Competitive binding of essential ions represents a newer antibacterial strategy. Released Cu^2+^ [32] or Zn^2+^ [42] ions compete with microbial uptake of Fe^3+^ and Mg^2+^ [42], disturbing key metabolic pathways. For example, nanometer zinc oxide (ZnONPs) combined with radio frequency (RF) heating can penetrate the surface of poultry meat to form a synergistic bactericidal effect while inhibiting lipid oxidation. Zn^2+^ releases iron carriers that competitively bind microbes, extending shelf life to more than 14 d [42]. This poultry-specific validation highlights the applicability of metal ion competitive chelation mechanisms in real chicken preservation scenarios.

Importantly, the adaptability of these antibacterial mechanisms differs substantially under the high-humidity conditions characteristic of poultry meat. Metal ion release systems can suffer from accelerated leaching and reduced control in moist matrices, requiring stabilization strategies such as mesoporous carriers or polyelectrolyte coatings to prevent uncontrolled migration. By contrast, ROS-based oxidative mechanisms often exhibit enhanced efficacy in humid environments due to increased availability of dissolved oxygen and water molecules that promote radical formation, although excess moisture may also lead to nonspecific oxidative reactions. Coordination-controlled release platforms, such as MOF- or polymer-based carriers, demonstrate superior stability by responding selectively to microbial metabolites rather than ambient moisture, thus maintaining targeted antimicrobial delivery. Membrane-disruption mechanisms relying on electrostatic interactions are less affected by humidity directly, but their efficiency may be compromised by competitive adsorption of water or proteins in poultry exudates. Taken together, these differences highlight that rational material design must balance humidity sensitivity with antibacterial robustness, favoring mechanisms that either exploit or withstand the high-water activity of poultry environments.

In summary, coordination chemistry-based antibacterial mechanisms encompass a rich spectrum of actions, from direct ion interference and ROS-mediated damage to intelligent antimicrobial delivery, membrane destabilization, and nutrient starvation. These mechanisms are complementary, and many advanced systems integrate 2 or more pathways to achieve synergistic effects. The main challenges that remain are the precise regulation of ion and ROS release to avoid adverse effects on meat quality, overcoming interference from complex poultry matrices rich in proteins and lipids, and ensuring biosafety for consumer use. Future efforts should focus on biodegradable carriers, tunable coordination bonds that respond to spoilage signals, and scalable fabrication methods to enable practical applications in poultry preservation.

## Freshness Monitoring via Coordination-Based Sensing

The accurate monitoring of poultry freshness is essential for food safety and supply chain transparency. Traditional indicators such as sensory evaluation, pH, or microbial counts are limited by subjectivity and time delay. In contrast, coordination chemistry-based sensing strategies enable rapid, sensitive, and real-time monitoring by exploiting selective metal–ligand interactions [43,44]. These systems convert molecular recognition into detectable optical, electrical, or catalytic signals, offering both laboratory-grade precision and the possibility of intelligent packaging integration (Table 2).

**Table 2.** Freshness monitoring via coordination-based sensing

Nanomaterial	Mechanism	Coordination	Detection target	Signal	Signaling mechanism	Carrier	Detection time	LOD	Linear interval	Reference
Au–PrFeO_3_	Coordination-induced chemical reactions altering the material structure	O_2_ coordination, coordination adsorption	H_2_S	Resistance change (Rg/Ra)	Semiconductor hole	Resistance sensor	28 s	10 ppb	0.01–1 ppm	[109]
HKUST-1 metal-organic framework	Metal–ligand coordination altering fluorescence/optical properties	NH_3_ coordination	NH_3_	Colorimetric	Protonation of organic dyes	Solid-state sensor	3 min	0.02 ppm	1–100 ppb, 0.1–1,340 ppm	[53]
MOF-5	Host–guest coordination adsorption, MOF/nanocage	Absorb	NH_3_	Fluorescence	Turn off fluorescent mode	Gas sensor	3 h	49.6 ppb	0–80.47 ppm	[110]
dsDNA-Cu NCs	Metal–ligand coordination altering fluorescence/optical properties	-NH_2_ coordination	Histamine	Fluorescence	Ratio fluorescence	Fluorescence spectrum	5 min	3.6 μM	7–55 μM	[47]
Graphene with chemical receptors, CRGE	Host–guest coordination adsorption, MOF/nanocage	Hydrogen bonding and electrostatic interactions	Putrescine and Cadaverine	Resistance change	Electrochemical impedance	Resistance sensor	130 s	7.29 ppb (PT); 27.04 ppb (CV)	7.29–11.88 ppb (PT); 27.04–38.09 ppb (CV)	[111]
La-Ce-MOF	Host–guest coordination adsorption, MOF/nanocage	Physical adsorption and hydrogen bonding	TMA, DMA, NH3, HCHO	Frequency variation of quartz crystal microbalance (QCM) (Δf)	Piezoelectric effect	QCM sensor	2 h	TMA: 0.16 μM DMA: 0.35 μM NH_3_: 0.94 μM HCHO: 1.42 μM	TMA: 3–45 μM DMA: 4–60 μM NH_3_: 7–105 μM HCHO: 6–90 μM	[51]
D-CNPs/G-QDs	Metal–ligand coordination altering fluorescence/optical properties	-NH_2_ coordination and electrostatic interaction	Spermine	Fluorescence	Ratio fluorescence	Smart phone	5 min	0.33 μM	2–40 μM	[50]
AuNPs and AgNPs	Coordination-induced nanoparticle aggregation/dispersion	Electrostatic adsorption	Spermine, SP; Spermidine, SD; Histamine, HS; Tryptamine, TP;	Colorimetric	SPR	UV–Vis	10 min	SP: 0.3 μM SD: 0.2 μM TP: 0.1 μM HS: 0.2 μM	SP: 0.1–10.0 μM SD: 0.1–10.0 μM TP: 0.1–10.0 μM HS: 0.1–10.0 μM	[45]
Ag-Zn NPs	Coordination-induced chemical reactions altering the material structure	-OH coordination	H_2_S	Colorimetric	Chemical reaction	Digital camera	0.5 h	2 mg/m^3^	2–50 mg/m^3^	[112]
Ag@MoS_2_ nanocomposites	Metal coordination catalysis in enzymatic reactions	Coordination adsorption	NH_3_	Resonance frequency (f)	Capacitance change	Split-ring resonator, SRR	18.4 s	<1 ppm	0.097 ppm^−1^	[56]

### Coordination-induced nanoparticle aggregation/dispersion

One of the most direct applications of coordination chemistry in freshness monitoring is the regulation of nanoparticle aggregation through analyte coordination. Target compounds such as biogenic amines and thiols can bridge or disrupt surface ligands, leading to dramatic optical shifts.

Gold and silver nanoparticles have been widely studied due to their strong localized surface plasmon resonance (LSPR). Abbasi-Moayed et al. [45] designed a dual AuNP probe that distinguished spermine from histamine via coordination-induced aggregation, achieving μM-level sensitivity with naked-eye color changes from red to blue. Similarly, AgNPs stabilized by d-ribose exhibited high selectivity toward methylamine released from spoiled chicken, producing a color shift from light yellow to deep red. Importantly, smartphone-based RGB (red, green, blue) analysis provided quantitative freshness evaluation, demonstrating the practicality of these systems in consumer-level monitoring [46].

To improve stability in humid poultry storage environments, researchers have developed polymer-coated NP (nanoparticle) probes. For example, polyvinyl alcohol (PVA)-coated AgNPs remained stable for 14 d at 4 °C while retaining sensitivity to cadaverine. By embedding such probes into packaging films, colorimetric changes could be observed without direct sample handling, highlighting the feasibility of “intelligent” poultry packaging [46].

Despite their simplicity and low cost, challenges remain in extending detection windows under high humidity and avoiding false positives from complex meat matrices. Integration with protective polymers and multiplexed readouts are promising solutions.

### Metal–ligand coordination altering fluorescence/optical properties

Fluorescent probes exploit coordination interactions to modulate emission intensity or wavelength. Compared to colorimetric methods, fluorescence provides higher sensitivity and multiplexing potential.

Copper nanoclusters (Cu NCs) are a prime example. dsDNA-stabilized Cu NCs undergo fluorescence quenching upon coordination with histamine, achieving detection limits of 3.6 μM. The selectivity derives from the preferential binding of imidazole groups to Cu^2+^ centers. Similarly, glutathione-stabilized Ag NCs display radiometric fluorescence shifts in response to putrescine, offering visual readouts with ppb (part per billion)-level sensitivity [47,48].

Lanthanide-based materials further expand this platform. Eu^3+^ and Tb^3+^ complexes coordinated with amino groups in biogenic amines show distinct radiometric emission changes, enabling visual freshness assessment. A recent lanthanide–nano-clay hybrid demonstrated stability under high humidity and a detection limit of 0.1 μM for cadaverine, making it well-suited for poultry monitoring [49].

Multicolor probes offer another dimension. CdTe quantum dots combined with molecularly imprinted polymers (MIPs) achieved 3-emission fluorescence for simultaneous detection of histamine, spermine, and putrescine. When coupled with machine learning, the system provided accurate classification of spoilage stages, demonstrating how coordination chemistry can be integrated with data science for next-generation monitoring [50].

### Host–guest coordination adsorption and MOF/nanocage

Metal–organic frameworks possess tunable pore sizes, large surface areas, and abundant coordination sites, making them powerful platforms for freshness sensing.

La–Ce MOFs were shown to detect trimethylamine with sub-μM sensitivity using quartz crystal microbalance. The high Lewis acidity of La^3+^ facilitates selective coordination with amine groups, resulting in measurable frequency shifts. Importantly, these sensors demonstrated rapid response and reusability after mild regeneration [51].

Hybrid MOF–plasmonic composites further improve sensitivity. Au@ZIF-8 sensors combine selective amine adsorption with surface-enhanced Raman scattering, amplifying signal intensity by over 10^4^-fold. Similarly, MOF–polymer films embedded in packaging exhibited continuous fluorescence quenching in response to volatile amines, providing both quantitative and visual readouts [52].

Beyond amines, MOFs can target aldehydes and sulfur compounds. Zn–BDC (1,4-benzenedicarboxylate) frameworks modified with thiol groups detected H₂S with a detection limit of 15 ppb, while Cu-based MOFs showed strong affinity toward formaldehyde, both of which are relevant spoilage markers in poultry [53,54].

The major advantages of MOF-based sensors are high selectivity, multifunctionality, and adaptability to complex environments. However, issues of water stability and food safety must be addressed before large-scale application.

### Coordination-induced chemical reactions altering the material structure

Some sensors harness coordination-triggered structural or electronic transitions to generate detectable signals, providing rapid and miniaturizable readouts.

For example, CuNi-MOF derivatives exposed to H₂S undergo Cu–S coordination and transformation into CuS, leading to resistance reduction measurable by simple conductometric devices. These sensors achieved ppb-level sensitivity and remained stable at refrigeration temperatures (4 °C), making them particularly attractive for cold chain poultry monitoring [55].

Ag@MoS₂ nanocomposites detect NH₃ through Ag–S coordination, resulting in resonance frequency shifts in wireless electronic circuits. Detection occurs within 20 s, enabling real-time, noninvasive monitoring. Other systems, such as polyaniline/MOF composites, exhibit colorimetric-to-electrical signal conversion, allowing dual-mode freshness evaluation [56].

These electrical approaches offer the advantage of integration into portable or wearable devices. When coupled with Bluetooth or NFC (near-field communication) technology, they enable Internet of Things (IoT)-based freshness monitoring across the supply chain. Future research should focus on ensuring sensor stability under fluctuating humidity and minimizing cross-sensitivity to CO₂ and water vapor.

### Metal coordination catalysis in enzymatic reactions

Nanozymes with coordination-engineered active sites can catalyze reactions that generate optical signals in response to spoilage compounds. This strategy mimics natural enzymes while offering superior stability.

Single-atom Fe–N₄ nanozymes catalyze peroxidase-like reactions, producing OH radicals that oxidize chromogenic substrates in the presence of biogenic amines. This results in dual colorimetric/fluorescence signals that correlate directly with poultry spoilage stages [57]. Similarly, Pt–CeO₂ heterojunction nanozymes accelerate electron transfer in xanthine oxidation, achieving detection limits as low as 1.2 μM [58].

In practical applications, such nanozymes have been embedded into polymer films for intelligent packaging. For example, a Fe–N₄–based film exhibited color changes from light yellow to deep blue as poultry freshness declined, with strong correlation to microbial counts and TVB-N levels. These systems not only monitor spoilage but also maintain long-term stability under refrigeration, addressing a key limitation of natural enzymes.

Coordination-based freshness monitoring provides a versatile platform that integrates colorimetric, fluorescence, MOF adsorption, electrical, and catalytic strategies, each offering distinct advantages such as low-cost visual detection through nanoparticle aggregation, ultra-sensitive quantification via fluorescence probes, selective adsorption and multifunctional detection by MOFs, seamless IoT integration through electrical responses, and durable dual-mode readouts enabled by nanozyme catalysis. To advance toward practical application, future efforts should focus on ensuring the biocompatibility and safety of sensing materials under food-contact conditions, enhancing matrix adaptability to maintain performance in humid and protein-rich poultry environments prone to interference, and achieving smart integration by coupling coordination sensors with wireless communication and machine learning to enable large-scale, real-time monitoring throughout the cold chain. Addressing these priorities will accelerate the translation of coordination chemistry-based sensing into practical deployment, ultimately providing consumers and industry stakeholders with reliable tools to safeguard poultry freshness and safety.

## Multifunctional Synergistic Effect of Nanomaterials in Poultry Preservation Strategies

**Fig. 4.**
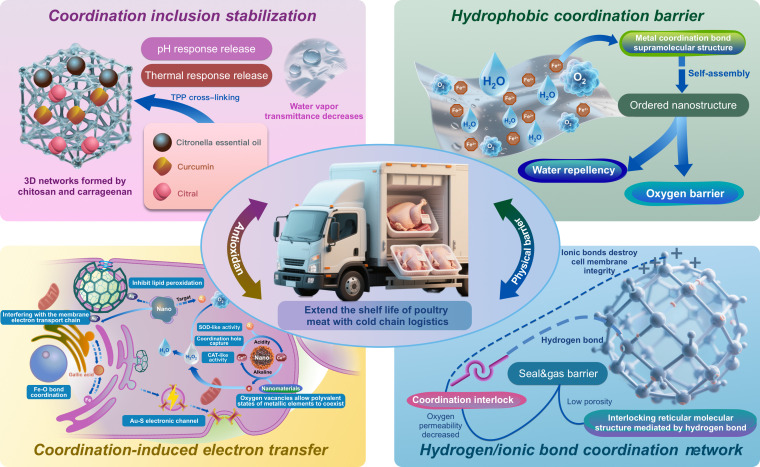
Multifunctional synergistic effect of nanomaterials in poultry preservation strategies.

The multifunctional synergistic effects of nanomaterials in poultry preservation strategies represent a paradigm shift in food packaging technology, leveraging coordination chemistry and nanomaterial engineering to address microbial proliferation, oxidative degradation, and moisture loss. Central to these advancements are 4 interrelated mechanisms: coordination inclusion stabilization, hydrophobic coordination barrier, hydrogen/ionic bond coordination network, and coordination-induced electron transfer (CIET). Collectively, these strategies integrate physical, chemical, and biological preservation pathways, demonstrating remarkable efficacy in extending shelf life, preserving sensory quality, and reducing spoilage-related losses in poultry products (Fig. 4 and Table 3).

**Table 3.** Preservation strategy based on coordination chemistry

Nanomaterial	Mechanism	Coordination	Food	Fresh-keeping effect	Carrier	Reference
TP-AgNPs	Hydrogen/ionic bond coordination network	-OH and -NH_2_ coordination	Food packing	Water vapor permeability decreased by 57.1%	CS film	[67]
NC	Hydrogen/ionic bond coordination network	-OH and -NH_2_ coordination	Pork minced meat	TBARs decreased to 0.736 mg MDA/kg on day 9	Chitosan aerogel	[61]
ZnO NPs and FeO NPs	Coordination-induced electron transfer​	Oxygen coordination	Chicken and lamb	Mutton shelf life is 15 d, chicken shelf life is 20 d	Biothermoplastic films based on starch	[113]
Nano-silicon	Hydrogen/ionic bond coordination network	Silicate coordination	Ajowan (*Trachyspermum ammi*)	The DPPH free radical scavenging ability was 4,126 μg/ml	Aqueous solution	[114]
AgNPs	Coordination-induced electron transfer	-NH_2_ and -SH coordination	Meat	50% reduction in TBARS (beef burger); 40% reduction in TBARS (chicken); 60% reduction in TBARS (camel minced meat);	PE and PLA	[115]
Nanoemulsions/nanocapsules	Hydrogen/Ionic bond coordination network	Ca^2+^ coordination	Wagyu and crossbred beef pastes	TBARS decreased by 50%	Sodium alginate and whey protein	[64]

### Coordination inclusion stabilization

Coordination inclusion stabilization represents a functional strategy for nanomaterials grounded in coordination chemistry principles. Its central concept is the construction of a stable nanocore structure via coordination between metal ions and organic ligands, enabling the selective encapsulation and controlled release of active substances. This mechanism primarily achieves preservation through 3 synergistic effects: orientation-driven assembly via coordination bonds, multistage encapsulation, and dynamic regulation of slow release.

Coordination bond-directed assembly is based on the formation of coordination bonds between surface active sites (amino, hydroxyl, etc.) in nano-callers such as chitosan, liposomes, and other nano-callers with metal ions such as Zn^2+^ and Fe^3+^ to construct a 3-dimensional network skeleton. In 2023, Gan et al. [59] coated lemongrass essential oil (LO) using chitosan-Carrageenan bilayer liposomes (C-CH-NL) in which chitosan (CH) was formed with sodium triphosphate (TPP) via electrostatic coordination of PO_4_^3−^ with NH₃ (NL). The zeta potential decreased from 38.2 mV to 22.5 mV. The coordination network had a sustained release protection effect on the volatile components of essential oils such as geranol and cital, and the inhibition rate of *Pseudomonas* in poultry meat was increased by 2.1 log CFU/g compared with that of free essential oils, and the shelf life was extended to 12 d. This demonstrates that coordination-stabilized essential oil systems are effective in poultry packaging, verifying their practical applicability.

The multistage envelop effect refers to the embedding of phenols, terpenes, and other active components into the coordination network through secondary interactions such as π–π stacking and hydrogen bonding. For example, Ji et al. [60] made the phenolic hydroxyl group of propolis extract (EEP) form a complex with phosphatidylcholine (PC) phosphate group, which increased the encapsulation rate of tea seed oil (TSO) nanocapsules to 89.6%, and the antioxidant activity was 1.8 times higher than that of free oil. In 2023, Zaharioudakis et al. [61] constructed a stable structure of 21-nm carvacrol nanoemulsion (NC) with a lecithin–casein coordination system, and its DPPH (2,2-diphenyl-1-picrylhydrazyl) clearance rate (92.4%) was 18.7% higher than that of microemulsion system (MC). After applying to ground pork, the TBARS value decreased by 63.2% compared with the control group, which confirmed that coordination embedding could slow down the oxidative deactivation of phenols.

In general, coordination bonds are pH/temperature responsive. Based on this property, targeted release of active substances can be achieved, that is, dynamic slow-release regulation. Shen et al. [62] loaded curcumin (CUR) through the chitosan–TPP coordination network, and the release rate of curcumin under pH 5.0 (meat putrefaction environment) was 3.2 times higher than that under neutral conditions, effectively inhibiting lipid peroxidation (TBARS value decreased by 46.3%). At the same time, the chitosan–propolis nanocomposite membrane constructed by Jia et al. forms a dense network via Zn^2^ bridging. Water vapor permeability (WVP) is reduced to 1.32 × 10^−10^ g·m^−1^·s^−1^·Pa^−1^, and a synergic inhibition of VBB-N (total volatile base nitrogen) in poultry meat is achieved (by 42.8%)[63]. It shows that the coordination structure regulates both physical barrier and chemical activity.

Coordination inclusion stabilization exhibits distinct advantages in the preservation of poultry meat. It enables precise control over the release of active substances via coordination bonds and is compatible with the synergistic effects of multiple natural components, including polyphenols, polysaccharides, and metal ions. Furthermore, the nanostructured system allows for a reduction in the required amounts of these active ingredients. This also helps to reduce fresh-keeping packaging costs. However, at the same time, this mechanism is greatly affected by the fat content of the food mechanism [64], and corresponding regulatory schemes should be designed for different poultry meat to ensure accurate and quantitative release. Future studies should focus on the development of biodegradable coordination systems such as Fe^3+^-polyphenol networks, as well as the long-term maintenance of coordination stability in cold chain conditions [62].

### Hydrophobic coordination barrier

The hydrophobic coordination barrier represents an innovative preservation strategy combining coordination chemistry with nanomaterial engineering. This mechanism involves the formation of supramolecular structures through metal–ligand coordination bonds, creating a hydrophobic interface that simultaneously inhibits microbial invasion and oxidative degradation. Coordination complexes typically consist of transition metal ions and organic ligands that self-assemble into ordered nanostructures that enable precise control of their hydrophobicity [65]. These nanostructures have 3 functions: a physical barrier against water vapor and oxygen penetration, sustained release of active ingredients through ligand bond dissociation, and targeted antibacterial activity through ligand–receptor interactions. The latter 2 functions have been mentioned above, and the blocking effect of physical barriers is mainly discussed in detail next.

In 2021, Kongkaoroptham et al. [66] constructed polyethylene glycol methyl methacrylate (PEGMA)- and deoxycholic acid (DC)-modified chitosan nanoparticles, which formed a stable coordination complex through -NH₂/Mg^2+^ interaction. The free radical scavenging activity of ABTS^+^ reached 85.5%. This ligation-induced hydrophobicity markedly improves moisture resistance (WVP is reduced by 38.7%) while keeping oxygen transport below 12 cm^3^/m^2^·d. Zhang and Jiang [67] demonstrated the double coordination mechanism in a 2021 case based on the antioxidant tea polyphenol biosynthesis of AgNPs. The catechol-O^−^/Ag^+^ coordination forms a pi–d electron conjugated system, while the terpene-Ag^+^ hydrophobic interaction forms a protective crown. This layered structure achieves slow-release kinetics (27.9% cumulative release over 9 d) while preventing nanoparticles from aggregating. The study by Liu et al. [68] provides direct evidence of this mechanism in the preservation of refrigerated pork. The cinnamaldehyde nanoemulsion (CNE) with the addition of Tween 80 emulsifier can form a ligand-stable oil droplet [diameter 94.37 nm, PDI (polydispersity index) 0.227], which forms a hydrophobic barrier at the meat surface interface. The noncontact preservation mode utilizes gaseous cinnamaldehyde release via ligand exchange reaction. The total microbial count was reduced by 1.82 log CFU/g compared to the control group, while the TVB-N value was maintained below 15 mg/100g for 7 d, and exudate loss was reduced by 42% compared to the direct contact method. Notably, coordinated mediated release features prevent flavor contamination while maintaining sensory acceptability. A similar mechanism was observed in the study by Kovačević et al. [69], where the thyme essential oil nanoemulsion inhibited *Salmonella* Typhimurium through hydrophobic interactions with membrane porins.

The mechanism of the hydrophobic coordination barrier offers 2 notable advantages in its current applications. First, the water barrier can bind oxygen and exhibit antioxidant activity. Second, the strength of the ligand–metal bond governs the release of active ingredients, which can be modulated through appropriate ligand selection. Nevertheless, challenges persist regarding industrial scalability and long-term stability. Future studies should focus on the development of biodegradable ligands while optimizing the environmental stability of the metal–ligand stoichiometry.

### Hydrogen/ionic bond coordination network

As an important interaction force in coordination chemistry, hydrogen and ionic bonds can inhibit both oxidation and microbial spoilage of fresh food by constructing dynamic network structures in the preservation mechanism of nanomaterials. The dynamic network structure has a molecular seal and gas barrier effect. The 3-dimensional network formed by hydrogen and ionic bonds of nanomaterials can markedly improve the density of packaging materials. For example, the amino group (-NH_2_) and hydroxyl group (-OH) on the chitosan molecular chain are self-assembled to form a continuous phase through hydrogen bonding, and the hydroxyl group of nanocellulose forms a cross-linked network with the chitosan side chain, which can effectively reduce the oxygen permeability [70,71]. In 2023, Rincón et al. [70] constructed chitosan–nanocellulose composite membranes and fixed antimicrobial peptides (Nisin) through hydrogen bond networks. The experimental results show that the porosity of chitosan aerogel containing 5% nanocellulose is reduced by 42%, and the water vapor barrier property is improved by 60%. This effect can be attributed to the strengthening of the material topology by hydrogen bond coordination between the nanofibers and chitosan. At the same time, the electrostatic interaction (ionic bond) between the cationic amino group and the phospholipid of the bacterial membrane can destroy the integrity of the membrane. Experiments showed that the material reduced the number of *Pseudomonas* in poultry meat by 2.3 log CFU/g.

At the same time, the hydrogen/ionic bond coordination network can also show antioxidant properties. Arnata et al. [72] made the phenol hydroxyl group of lignin nanoparticles form an electron transport network through hydrogen bonding, which can efficiently remove DPPH and ABTS free radicals. Nano-treatment increased the specific surface area of lignin and the exposure of the phenol hydroxyl group, and its antioxidant capacity reached 1.5 times that of BHT (butylated hydroxytoluene). A similar mechanism can also be seen in the lauric-supported system of shell aerogel studied by Rincón et al. [70], in which the ionic bond between carboxylic acid and chitosan protonated amino group enhances the inhibition effect of lipid peroxidation. At the same time, the hydrogen bond network of lignin nanoparticles adsorbs metal ions such as Fe^2+^ in poultry exudate, inhibiting the Fenton reaction. Packaging containing 2% nano-lignin can control the proportion of high iron myoglobin in poultry meat below 20% and extend the shelf life to 10 d [72].

The hydrogen- and ionic-bond coordination network enhances the barrier, antibacterial, and antioxidant properties of nanocomposites through molecular interface engineering, offering an efficient strategy for the preservation of fresh poultry meat. Future research should prioritize the dynamic design of coordination bonds to achieve intelligent, controlled-release functionality. Additionally, the synergistic coordination mechanisms between lignin nanoparticles and the TiO₂–chitosan system require further investigation to facilitate their integrated application in active packaging.

### Coordination-induced electron transfer

CIET is one of the core mechanisms for metal-based nanomaterials to exert antioxidant activity, and its essence is to regulate electron transport paths through coordination between metal centers and ligands. Thus, efficient removal of ROS can be achieved. This mechanism has important application value in nano-antioxidant and nano-enzyme systems. Take the example of CeO₂ nanoparticles; the presence of oxygen vacancies on their surface leads to the coexistence of Ce^3+^/Ce^4+^ mixed valence states. Changes such as the coordination environment, like modifications of surface hydroxyl groups or organic ligands, can markedly affect the ability of Ce^3+^/Ce^4+^ to REDOX cycle [73]. Under alkaline conditions, Ce^3+^ transfers electrons to H_2_O_2_ by coordination action, catalyzing its decomposition into H₂O and O₂, i.e., cat-like activity. In an acidic environment, Ce^4+^ achieves SOD (superoxide dismutase)-like activity by trapping the electrons of the superoxide radical (O₂^−^) through a coordination hole [74]. This dynamic coupling of valence transition and coordination structure forms the chemical basis of CIET.

MOF materials are typical carriers of the CIET mechanism. For example, CE-MOF takes 4,4′,4″-nitrotribenzoic acid as an organic ligand and forms a porous structure to expose more active sites by regulating the strength of the Ce^3+^ coordination with carboxylic acid groups. The π–π-conjugated system of ligands can promote electron delocalization and accelerate the capture and neutralization of ROS. Similarly, in the Au/Ce shell nanostructure, n-acetylcysteine and other sulfhydryl ligands on the surface of Au form electron transport channels through the sulfur–gold bond, which increases the SOD activity of Au nanoparticles by 10^4^ times [74]. At the same time, PEG or amino acid modification can change the surface charge distribution of nanoparticles, thereby regulating their interaction with ROS. For example, PEG-coated CeO nanoparticles enhance their coordination adsorption with H_2_O_2_ by forming a hydrophilic shell, and their CAT (catalase)-like activity is 4.2 times higher than that of unmodified samples. In addition, Fe_3_O_4_ nanoparticles were functionalized with gallic acid. The coordination of the phenol hydroxyl group with Fe^3+^ substantially increased their ability to scavenge DPPH radicals [74].

In the preservation of fresh poultry meat, ligation-induced electron transfer can be divided into 3 functional categories: antioxidant and bacteriostatic coordination, pH intelligent response, and multi-enzyme coordination. AgNPs release Ag^+^ through surface coordination, interfering with the electron transport chain of microbial cell membranes [73]. Meanwhile, the antioxidant activity of AgNPs can inhibit lipid peroxidation [75]. For example, AgNPs synthesized from plant extracts can simultaneously reduce TBARS values and *E. coli* counts in poultry meat [76]. In terms of intelligent response, pH-responsive CeO nanoparticles can automatically enhance SOD-like activity and target O_2_^−^ in the acidic microenvironment generated by poultry meat corruption. Studies have shown that the active packaging film loaded with CeO₂ can prolong the storage period of chicken by 40% and reduce the growth rate of TVB-N by 62% [74]. In terms of multi-enzyme synergism, the MN₃O₄ nanoflower prepared by Singh et al. [77] exhibited SOD, CAT, and GPin-like activities. Their porous structure adsorbed GSH (glutathione) through coordination, enabling a cascade of H₂O₂→H₂O. When used in poultry packaging, it can simultaneously inhibit protein oxidation and microbial proliferation, reduce carbonyl content by 55%, and reduce the total number of colonies by 3 log CFU/g.

The ligation-induced electron transfer mechanism provides an efficient and controllable technological path for fresh poultry preservation by precisely regulating the surface chemical environment of nanomaterials. In the future, it is necessary to further explore the dynamic response mechanism of coordination bonds, develop environmentally friendly ligand systems, and establish a safety evaluation model of nanomaterial migration law.

In summary, the multifunctional synergy of nanomaterials in poultry preservation hinges on the strategic exploitation of coordination chemistry principles, enabling tailored interactions between nanostructured matrices and bioactive components. The discussed mechanisms—coordination inclusion stabilization, hydrophobic coordination barrier, hydrogen/ionic bond networks, and CIET—collectively enhance preservation outcomes through spatially and temporally controlled release of antimicrobials, ROS scavenging, and barrier optimization. However, challenges persist in scaling up production, ensuring long-term stability under cold chain conditions, and addressing potential nanomaterial migration risks. Future research must prioritize the development of biodegradable coordination systems, dynamic ligand-responsive designs, and comprehensive safety assessments to bridge laboratory innovations with commercial viability. By advancing these areas, nanotechnology-driven preservation strategies will not only meet the growing demand for sustainable food packaging but also set new benchmarks for quality and safety in the poultry industry.

## Evolution of SMART Packaging for Poultry Meat Products

The development of smart packaging systems for meat products has progressed through distinct technological phases, propelled by interdisciplinary advances in materials science, sensing technologies, and digital integration. Early implementations concentrated on basic freshness indicators, including pH-responsive colorimetric sensors derived from natural pigments such as anthocyanins and curcumin [78]. These systems utilized biopolymer matrices to detect volatile amines from microbial spoilage through visible color changes [79]. However, limited stability under variable storage conditions and inadequate quantification capabilities constrained their adoption in commercial supply chains.

The emergence of nanotechnology-enabled active packaging marked a critical transition. Metal nanoparticles (Ag, ZnO) and nanoemulsions were incorporated into biopolymer films to confer antimicrobial properties while maintaining biodegradability [80]. For instance, zinc oxide nanoparticles demonstrated dual functionality by inhibiting microbial growth and interacting with anthocyanin-based indicators to enhance spoilage detection sensitivity. This phase emphasized material hybridization, where cellulose nanofibrils and polylactic acid composites improved mechanical strength and gas barrier properties while embedding pH-sensitive natural dyes [81].

### The combination of coordination nanomaterials and packaging

The incorporation of coordination nanomaterials into packaging matrices has emerged as an advanced strategy to enhance both the structural and functional performance of smart packaging. Such nanomaterials, including metal–organic frameworks and cellulose nanocrystals, are typically embedded within biopolymer matrices via electrostatic interactions, covalent bonding, or physical encapsulation. For example, cellulose-based hydrogels functionalized with anthocyanins or curcumin exploit hydrogen bonding and π–π stacking to stabilize pH-sensitive pigments within the polymer network, thereby enabling real-time colorimetric responses to spoilage-related metabolites such as ammonia or biogenic amines. Similarly, Pickering emulsions stabilized by laurate esterified starch nanoparticles have been used to encapsulate curcumin, where the nanoscale interfacial architecture ensures controlled release and improved oxidative stability of the indicator [82]. Such integration not only enhances mechanical robustness and barrier properties but also ensures uniform dispersion of active components, critical for consistent freshness visualization. Furthermore, the leaching of nanoparticles into food matrices raises concerns about regulatory compliance and consumer safety, necessitating advanced encapsulation methods or covalent immobilization strategies to mitigate migration risks.

### Convergence with digital technologies

The integration of RFID and IoT transformed smart packaging into networked food safety systems. Ultra-high frequency (UHF) RFID tags printed on biodegradable cellulose substrates enabled wireless temperature/humidity tracking throughout cold chain logistics [83]. Chipless RFID sensors utilizing nanomaterial-enhanced capacitive resonators achieved multi-parameter detection (pH, gas composition, pressure) without battery dependence [84]. These systems bridged physical packaging with cloud-based analytics, exemplified by smartphone-readable QR codes that linked packaging sensors to blockchain-tracked freshness data [19,85].

However, critical challenges persist: Nanomaterial migration risks in acidic meat matrices require rigorous biocompatibility assessments [86]. Besides, multi-tag interference in RFID-enabled pallet-scale monitoring reduces data reliability [87]. Moreover, consumer acceptance barriers for color-changing indicators necessitate standardized interpretation guidelines [88].

Looking ahead, the integration of nanomaterial-enabled sensors with RFID and blockchain platforms is poised to move beyond pilot trials and into routine cold chain logistics within the next 5 to 10 years, enabling real-time freshness verification, automated shelf-life management, and transparent traceability across poultry supply chains. Such developments are expected to transform current industry practices by reducing waste, lowering safety risks, and building consumer trust through data-driven packaging solutions.

While advancements in coordination nanomaterials and bio-based matrices have propelled smart packaging toward multifunctionality, several limitations hinder large-scale adoption. First, the scalability of nanomaterial synthesis remains constrained by high costs and complex fabrication processes. For instance, MOF-based sensors require precise control over crystallization conditions, which complicates industrial production [87]. Second, the environmental impact of nanomaterial recovery and biodegradability is underexplored. Although cellulose and starch derivatives are marketed as sustainable, their nanocomposites often incorporate nondegradable synthetic polymers or metals, undermining circular economy principles [89,90].

Beyond laboratory-scale demonstrations, several pilot studies and industrial trials illustrate the feasibility of integrating RFID and blockchain in meat and poultry supply chains. For instance, screen-printed UHF RFID tags on cellulose substrates have already been applied in pilot cold chain logistics to provide real-time temperature tracking with smartphone readability, enabling small-scale producers to access cloud-based freshness data during transport. Similarly, chipless RFID sensors enhanced with nanomaterial-based resonators have been tested in pallet-level monitoring of packaged meat, successfully transmitting humidity and gas composition data without battery dependence, although multi-tag interference remains a challenge. On the blockchain side, smartphone-readable QR codes linked to packaging sensors have been explored in fruit and meat supply chains, where each transaction is immutably recorded, thereby reducing fraud and enhancing consumer trust. These examples demonstrate that combining RFID-enabled sensing with blockchain-backed traceability not only ensures transparency in quality monitoring but also provides actionable data for retailers and regulators to validate shelf-life claims. Expanding such pilot initiatives to large-scale poultry systems will be critical for realizing the full potential of digitally integrated smart packaging. Nevertheless, industrial implementation still faces important barriers. High initial investment and operating costs may limit adoption by small- and medium-sized enterprises. Regulatory hurdles, including compliance with food safety authorities and data protection frameworks, can further slow down deployment. Moreover, consumer acceptance remains uncertain, as while transparency may increase trust, it may also raise privacy concerns and cost sensitivity. These practical considerations must be addressed before blockchain-enabled traceability can achieve full-scale industrial adoption in poultry packaging.

Future research must prioritize the development of self-adaptive systems that integrate multi-parameter sensing with machine learning algorithms for predictive freshness modeling. For example, hybrid platforms combining RFID-based humidity sensors with colorimetric indicators could enable dual-mode monitoring, enhancing reliability in dynamic supply chains. Additionally, the use of bio-inspired designs—such as mussel-adhesive polymers or enzyme-mimetic nanomaterials—could improve interfacial adhesion between sensors and packaging matrices, reducing signal drift. Regulatory frameworks must also evolve to address nanoparticle toxicity and lifecycle impacts, ensuring that “green” claims align with material realities [91].

In conclusion, the convergence of nanotechnology, biomimetics, and digital integration holds transformative potential for smart packaging [92]. However, overcoming technical, economic, and ecological barriers will require interdisciplinary collaboration and a critical reassessment of sustainability metrics beyond mere biodegradability claims.

## Summary and Conclusion

This review establishes a transformative framework for intelligent poultry preservation through the integration of coordination chemistry principles with nanomaterial engineering and digital food systems. The proposed paradigm demonstrates unprecedented potential in addressing poultry-specific spoilage challenges by synergizing 3 critical functionalities: multi-target antimicrobial action, dynamic freshness monitoring, and microenvironment regulation. Coordination-driven nanomaterials exhibit superior preservation efficacy through programmable metal–ligand interactions that enable pH-responsive antimicrobial release, ROS-mediated oxidative damage, and competitive nutrient chelation, effectively countering poultry’s biochemical vulnerabilities including high water activity and lipid oxidation susceptibility. The inherent molecular recognition capabilities of coordination complexes further empower real-time freshness assessment through biomarker-responsive optical/electrochemical signaling, overcoming traditional packaging’s limitations in quality visualization.

Despite these advancements, critical challenges persist in material stability under high humidity conditions, potential ion migration risks, and scalability of nano-functionalized packaging fabrication. The complex poultry matrix introduces interference risks through competitive ligand binding with endogenous proteins and lipids, potentially compromising both antimicrobial efficiency and sensing accuracy. To address these limitations, future research should prioritize the development of bioinspired coordination networks with self-healing capabilities and edible matrix compatibility. Hierarchical material architectures combining MOF-based oxygen scavengers with humidity-regulating hydrogels could achieve microenvironmental stabilization through coordinated gas/water sorption mechanisms, while covalent organic framework (COF)-enhanced barrier films may suppress lipid oxidation through π–π stacking interactions with unsaturated fatty acids.

The convergence of coordination-engineered nanomaterials with advanced packaging structural design presents new opportunities for system-level optimization. Oxygen-scavenging films with graded porosity architectures can synergize with MOF-embedded humidity buffers to create adaptive preservation microenvironments. Emerging digital integration strategies bridging molecular-scale sensing with macroscale supply chain management coordination-responsive indicators coupled with QR code/RFID tags enable blockchain-compatible traceability through machine learning-enhanced spoilage prediction models. This digital–physical fusion allows real-time quality mapping via wireless sensor networks, where nanomaterial-generated chemical data inform dynamic preservation parameter adjustments throughout cold chain logistics. Future systems could implement edge computing-enabled smart packaging that autonomously modulates antimicrobial release profiles based on accumulated temperature history and biomarker detection patterns.

Beyond technological innovation, the safety of coordination-engineered nanomaterials in food-contact applications remains a decisive factor for translation into practice. Migration behavior is a central concern, as nanoparticles or metal ions may leach from packaging matrices into poultry meat under acidic, high-humidity, or lipid-rich conditions, potentially altering microbial dynamics or posing unintended toxicological effects. Existing studies highlight that the extent of migration depends on factors such as particle size, surface modification, and matrix compatibility, with ionic forms often exhibiting higher mobility. These behaviors necessitate systematic assessments of long-term stability and release kinetics in real food environments. Toxicological risks are further amplified by the possibility of ROS generation or competitive binding with endogenous biomolecules, raising concerns about oxidative stress and bioaccumulation. Addressing these issues requires rigorous in vitro and in vivo evaluations, including chronic exposure models, to clarify dose–response relationships and establish safety thresholds. Importantly, regulatory frameworks such as the European Food Safety Authority (EFSA) guidelines for food-contact nanomaterials and the U.S. Food and Drug Administration (FDA) regulations emphasize the necessity of migration testing, toxicological risk assessment, and lifecycle analysis before approval. However, specific standards for coordination-based nanomaterials are still evolving, highlighting the urgent need for harmonized international protocols. Strengthening these regulatory perspectives will ensure that future smart packaging solutions not only achieve technical efficacy but also meet the fundamental requirements of consumer protection and public health.

To realize this vision, interdisciplinary efforts must address critical knowledge gaps in nanomaterial–digital interface development, including standardized data protocols for coordinating sensor outputs and cybersecurity in blockchain implementations. Scalable manufacturing requires breakthroughs in roll-to-roll nano-coating technologies and artificial intelligence (AI)-optimized material formulations. Environmental considerations demand lifecycle assessments of coordinated nanomaterials and closed-loop recycling strategies for smart packaging components. By bridging molecular precision with systems engineering, this coordination chemistry paradigm promises to revolutionize poultry preservation through intelligent, self-regulating platforms that simultaneously ensure food safety, reduce waste, and enable transparent supply chains, ultimately contributing to sustainable protein production in the era of digital agriculture.

In parallel, the digital integration of RFID and blockchain with nanomaterial-based packaging is likely to enter industrial deployment within the next decade, representing a realistic pathway for reshaping poultry preservation practices through enhanced transparency, automation, and consumer confidence.

Looking further ahead, coordination-driven nanomaterials hold the potential to influence not only the extension of poultry shelf-life but also the broader evolution of global standards in food safety and sustainability. By aligning advanced packaging strategies with international regulatory frameworks and sustainable development goals, these materials could serve as a model for next-generation food systems that prioritize both consumer health and environmental stewardship.

In conclusion, while coordination-driven nanomaterials offer transformative potential for poultry preservation, their widespread application depends on overcoming several urgent scientific challenges. Key priorities include establishing rigorous biosafety evaluations for nanomaterial migration in complex food matrices, developing scalable and cost-effective manufacturing processes, and building regulatory frameworks that standardize safety, efficacy, and consumer acceptance. Addressing these issues will be essential to bridge laboratory innovations with industrial-scale implementation and ensure that smart packaging systems achieve both sustainability and public trust.

## Data Availability

All data that support the findings of this study are reported in the main text. Source data are available from the corresponding authors on reasonable request.
